# Risk of intracranial hemorrhage in users of oral antithrombotic drugs: Study protocol for a nationwide study

**DOI:** 10.12688/f1000research.7633.1

**Published:** 2015-12-30

**Authors:** Sasha Gulati, Ole Solheim, Sven M. Carlsen, Lise R. Øie, Heidi Jensberg, Agnete M. Gulati, Charalampis Giannadakis, Asgeir S. Jakola, Øyvind Salvesen

**Affiliations:** 1 Department of Neurosurgery, St. Olavs University Hospital, Trondheim, 7030, Norway; 2 Department of Neuroscience, Norwegian University of Science and Technology (NTNU), Trondheim, 7491, Norway; 3 National Advisory Unit on Spinal Surgery, St. Olavs University Hospital, Trondheim, 7030, Norway; 4 Norwegian Centre of Competence in Deep Brain Stimulation for Movement Disorders, St. Olavs University Hospita, Trondheim, 7030, Norway; 5 National Advisory Unit on Ultrasound and Image-Guided Therapy, St. Olavs University Hospital, Trondheim, 7030, Norway; 6 Department of Endocrinology, St. Olavs University Hospital, Trondheim, 7030, Norway; 7 Unit for Applied Clinical Research, Department of Cancer Research and Molecular Medicine,, Norwegian University of Science and Technology (NTNU), Trondheim, 7491, Norway; 8 Department of Neurology, St. Olavs University Hospital, Trondheim, 7030, Norway; 9 Norwegian Patient Registry, Trondheim, 1601, Norway; 10 Department of Rheumatology, St. Olavs University Hospital, Trondheim, 7030, Norway; 11 Department of Neurosurgery, Sahlgrenska University Hospital, Gothenburg, 413 45, Sweden; 12 Institute of Neuroscience and Physiology, University of Gothenburg, Sahlgrenska Academy, Gothenburg, 413 90, Sweden

**Keywords:** Stroke, Hematoma, Drug-Related Side Effects and Adverse Reactions, Hematoma, Subdural, Intracranial

## Abstract

**Background **A wide range of antithrombotic medications can be used in the prevention and treatment of thrombosis. Among hemorrhagic complications of antithrombotic drugs, intracranial hemorrhage may have particularly devastating consequences with high morbidity, disability and mortality rates. The incidence and risks of intracranial hemorrhage in patients on antithrombotic treatments from regular clinical practice outside clinical trials remain largely unknown. It is not known if results from clinical trials can be extrapolated to everyday clinical practice. We will conduct a nationwide study to investigate the risks and incidence rates of intracranial hemorrhage in users oral antithrombotic drugs in Norway from 2008 through 2014.
** **

**Methods and design **The aim of this nationwide study is to investigate the incidence rates of intracranial hemorrhage requiring hospitalization in users of oral antithrombotic drugs. The study will be conducted within the approximately 4.7 million inhabitants of Norway from January 1 ^st^, 2008, to December 31 ^st^, 2014. Treatment and outcome data are obtained from the Norwegian patient registry and the Norwegian prescription database.
** **

**Trial registration number **Clinicaltrials.gov (NCT02481011)

## Background

According to the World Health Organization (WHO) the disorders caused by thrombosis are collectively the most frequent cause of death and disability in the developed world ^1^. A wide range of antithrombotic medications can be used in the prevention and treatment of thrombosis. There are two main groups of antithrombotic therapy: anticoagulants which limit activity of the coagulation cascade, and antiplatelet agents which limit activation or activity of blood platelets ^2^. Certain disorders are best managed with antiplatelet medications, others with anticoagulants, and some with both ^2^.

The challenge is to prevent thrombosis while maintaining hemostasis, namely the capacity to preclude hemorrhage. The most serious adverse effect of antithrombotic therapy is bleeding. Combinations of antithrombotic agents are now frequently used, and this may lead to an increased frequency of significant bleeding complications ^3– 6^. Among hemorrhagic complications of antithrombotic drugs, intracranial hemorrhage (ICH) may have particularly devastating consequences with high morbidity, disability and even mortality rates ^7, 8^. Intracerebral hemorrhage is generally associated with a higher risk for death and incurs greater loss of health over a lifetime than ischemic stroke ^9– 11^.

Although a certain risk for bleeding may be acceptable in the context of even greater protection against ischemic events, it is important to quantify the magnitude of bleeding risk. So far the efficacy and safety profile of antithrombotic agents are generally assessed in randomized controlled trials (RCT). However, extrapolating the results from RCTs to the general patient population in this context is challenging. Patients who participate in clinical trials are frequently highly selected and may therefore not be representative of users in everyday clinical practice. Clinical follow-up and drug compliance are often better in clinical trials and polypharmacy is less common than in clinical practice. In addition, the primary endpoint of RCTs is usually not complications, and complication numbers are limited as treatment period is often much shorter than in routine management of a chronic disease or condition. In everyday practice there is a risk of drifts in indications, inclusion criteria, exclusion criteria, and stop-criteria suggested by RCTs. Specifically, in the context of antithrombotic drugs this may potentially lead to treating patients with lesser risk of thromboembolic events but with higher risk of hemorrhage. Collectively these factors may lead to other incidence rates in the general clinical population than what is frequently reported in the literature.

The incidence of intracranial hemorrhage due to antithrombotic therapy could theoretically be monitored by post-marketing surveillance by including spontaneously reported events. Unfortunately, it seems this does not provide more reliable estimates. A recent study from Finland showed that bleeding complications due to oral anticoagulation with Warfarin are underreported in daily clinical practice ^12^. Further, it has been shown that reporting rates of side effects following medical therapy tend to decrease over time indicating that it is more likely that adverse events to a newer drug are reported compared to a drug that has been available for many years ^13, 14^. This is why we need population-based large-scale pharmaco-epidemiological studies, i.e. real world data, in which cohorts of patients exposed to antithrombotic medications are monitored to estimate a valid and reliable risk of the treatment.

The incidence rates of intracranial hemorrhage in patients on antithrombotic treatments in the general population remain unknown. We will conduct a nationwide study to investigate the incidence rates of ICH in users oral antithrombotic drugs in Norway from 2008 through 2014.

## Methods and design

Reporting will be consistent with the strengthening the reporting of observational studies in epidemiology (STROBE) statement.

### Aims of the study

The primary aim of this nationwide study is to investigate the incidence rates of ICH requiring hospitalization (inpatient treatment) in users of different oral antithrombotic drugs. Secondary aims are assessments of case-fatality rates following ICH and proportion of patients undergoing neurosurgical procedures in users and non-users of antithrombotic drugs.

### Study population

The study will be conducted within the approximately 4.7 million inhabitants of Norway (2008 census, Statistics Norway) from January 1 ^st^, 2008, to December 31 ^st^, 2014. All residents included in the Norwegian patient registry (NPR) and/or Norwegian prescription database (NorPD) aged 18 years or older at the start of the study are eligible for inclusion. According to NPR, the expected number of eligible patients with ICH during the study period is in the range of 50,000 to 60,000. The National Registry provides information to NPR and NorPD on vital status (dead or alive). Information about public specialist health care (both inpatient and outpatient treatment) and any dispensed prescription in Norway can be linked to residents by using a unique 11-digit personal identifier.

### Inclusion criteria

1. Hospitalization due to ICH ( Table 1)
2. Residential address in Norway in the entire study period
3. Age ≥18 years on January 1 ^st^ 2008

**Table 1.** The ICD-10 and ICPC-2 groups of ICH and comorbidity screened in this study.

Diagnoses	ICD-10	ICPC-2
**Intracranial hemorrhage**		
Subarachnoid hemorrhage Intracerebral hemorrhage	I60.0–I60.9 I61.0–I61.9	
Nontraumatic intracranial hemorrhage	I62.0–I62.9	
Intracranial injury	S06.3–S06.9	
**Comorbid conditions**		
Atrial fibrillation	I48	K78
Thromboembolism	I63–I68, I20–125, I74, G45.8, G45.9	K93, K94
Vascular disease	I21, I22, I70.0, I70.2–I70.9, F01	K74, K75, K76, K89, K90, K91, K92, K99
Alcohol abuse	G31.2, G62.1, G72.1, I42.6, K29.2, K70, K86.0, O35.4, T51, Z71.4, Z72.1	
Liver disease	B15–19, C22, D68.4, K70–K77, Z94.4	
Osteoarthritis	M19	
Peptic ulcer	K25–K29	
Diabetes mellitus	E10–E14	T89, T90
Hypertension	I10–I15	K85, K86, K87
Heart failure	I11.0, I42, I50, J81	K77, K82, K83, K84
Chronic renal failure	E10.2, E11.2, E13.2, E14.2, I12.0, N00–N08, N11, N12, N14, N17–N19, N26, N15.8–N16.0, N16.2–N16.4, Q61	
Bleeding	I69.0–I69.2, J94.2, K25.0, K25.4, K26.0, K26.4, K27.0, K28.0, K92.0–K92.2, N02, R04, R31	
**Accidents**	V0n–Y3n, S06.0–S06.3, S10–T19	

ICD-10 = 10 ^th^ revision of the International Statistical Classification of Diseases and Related Health Problems ICPC-2 = version 2 of the International Classification of Primary Care

### Exclusion criteria

1. Traumatic (high-energy) intracranial injury
2. Parenteral antithrombotic treatment as this information is not retained in NorPD

### The Norwegian health care system

Norway has a public health care system with quite evenly distributed resources and uniform training and licensing for medical professionals. Only public hospitals provide health care to patients with ICH. The health authorities cover all inpatient treatment for patients with intracranial hemorrhage, and costs concerning established treatment options are generally not a concern for the individual patients or their doctors. Further, the government covers a variable proportion of the costs of antithrombotic drugs prescribed by physicians. Preapproved medicines available for general reimbursement ensure that patients get part of the medicine expenses covered by the government when having a chronic, prolonged or severe illness. This ensures access to medicines regardless of financial situation. There are in general few disparities in access to health care in the Norwegian population and insurance policies do not influence the treatment of intracranial hemorrhage. Antithrombotic medicine in Norway is only available at pharmacies if the patient has a prescription from a physician.

### The Norwegian prescription database (NorPD)

NorPD was initiated on January 1 ^st^ 2004, and contains important information on prescriptions for medications dispensed from all pharmacies throughout the country. All pharmacies are required to register each drug dispensing in NorPD, ensuring complete registration. NorPD registers the unique personal identification number of the patient, type of drug according to the Anatomical Therapeutic Chemical (ATC) classification system, number of Defined Daily Doses as defined by WHO, date of dispensing, quantity dispensed, and drug strength and formulation. NorPD does not include the prescribed daily dose. In addition, diagnoses are registered for medications with reimbursement according to the 10 ^th^ revision of the International Statistical Classification of Diseases and Related Health Problems (ICD-10) or version 2 of the International Classification of Primary Care (ICPC-2). The 11-digit personal identification number, which is encrypted in NorPD, ensures that a complete prescription history can be established for each individual. The oral antithrombotic drugs included in the present study are presented in Table 2. The number of patients on oral antithrombotic drugs, either single agent or combined regimens) during the decade-long study period will be retrieved from NorPD.

**Table 2.** Oral antithrombotics and concomitant medication included in this study.

**ATC#**	
	**Antiplatelet agents**
**B01AC06**	Acetylsalicylic acid
**B01AC07**	Dipyridamole
**B01AC04**	Clopidogrel
**B01AC30**	Dipyridamole-acetylsalicylic acid
**B01AC22**	Prasugrel
**B01AC24**	Ticagrelor
**B01AC05**	Tiklopidin
	**Anticoagulants**
**B01AA03**	Warfarin
**B01AE07**	Dabigatran
**B01AF02**	Apiksaban
**B01AF01**	Rivaroksaban
**B01AA01**	Dikumarol
**B01AA02**	Fenylindandion
	**Concomitant medication**
**M01A**	NSAID
**C10A**	Statin
**C09**	Renin-angiotensin system inhibitor
**C03C**	Loop diuretic
**C03A**	Thiazide
**C07, C08,** **C01AA05,** **C01AA04,** **C01BD01,** **C07AA07,** **C01BC**	Antiarrhythmic drug
**A02BC**	Proton-pump inhibitor
**A10**	Oral glucose-lowering drug
**N06A**	Antidepressants
**H02AB**	Glucocorticoid

ATC = Anatomical Therapeutic Chemical classification system

### The Norwegian patient registry (NPR)

NPR automatically receives information regarding diagnoses and procedures when patients receive both inpatient and outpatient treatment by public Norwegian specialist health care services. The information required in the present study is available from January 1 ^st^ 2008. The ICD-10 subgroups of ICH that will be screened for inclusion are presented in Table 1. We will determine the incidence rates of ICH in users and non-users of oral antithrombotic treatment by linking data from NPR and NorPD.

### Comorbidity and concomitant medication

We will identify comorbidities from both NPR and NorPD, including atrial fibrillation, congestive heart failure, thromboembolism, vascular disease, hypertension, diabetes mellitus, peptic ulcer, liver disease, alcohol abuse, osteoarthritis, previous bleeding, and chronic renal failure. Dispensed prescriptions for renin–angiotensin system inhibitors, antiarrhythmic drugs (beta-blockers, digoxin, class 1C antiarrhythmic drugs, calcium-channel blockers, and amiodarone), non-steroidal anti-inflammatory drugs, antidepressants, or proton pump inhibitors dispensed are defined as concomitant medication.

### Statistics

All statistical analyses are performed with SPSS 21.0, MySQL (Oracle), or R version 3.1 (R Foundation for Statistical Computing). The statistical significance level is defined as P≤0.05 with no adjustments for multiple comparisons. For each patient exposure periods for antithrombotic medications will be calculated according to the World Health Organization’s recommendation for drug utilization studies using the ATC classification system and the DDD as a measuring unit ^15^. Exposure is defined as having occurred when patients have drug available and discontinuation as when they have no more drug(s) available. For many patients, treatment regimens are expected to change during the study period, so we treat use of antithrombotic medications in the analyses as time varying exposures. Consequently, patients can change exposure group according to dispensed prescriptions during the entire span of the study period. We consider patients to be at risk only when exposed to the drug(s) (during active treatment). We calculate risk time (person years) only for the active treatment period. Patients are followed until death, emigration or end of study period. Drug exposure and registration of comorbidity are discontinued (censored) at the time of the first event after 2008 (ICH). Due to strict Norwegian data privacy regulations the exact dates for prescription dispensing, ICH, and death are not made available to the study authors, and all time measurements are from a reference date known only to NPR and NorPD. The calendar year for ICH is available. The month and calendar year for death is available. Incidence rates will be calculated and compared between users and non-users of antithrombotic drugs for overall risk of intracranial hemorrhage and for the subgroups non-traumatic intracerebral hemorrhage, acute or chronic subdural hematoma, and subarachnoid hemorrhage. We will estimate hazard ratios with 95% confidence intervals for ICH using Cox regression models with adjustments for age, sex, concomitant drugs, and comorbidity. The time variable in the Cox model is patient age. We will investigate case fatality rates at three-month and one-year timepoints in addition to an analysis of overall survival following ICH. Differences in time to event (death) will also be presented in survival curves. We will investigate the proportions of patients with ICH undergoing neurosurgical procedures for different antithrombotic drug exposures. We will analyze continuous variables using an unpaired two-tailed t test for normally distributed data and continuous data with skewed distribution using the Mann-Whitney U test. The Chi-square test is used to examine the associations between categorical variables. For all outcome measures the statistician (ØS) will be blinded to drug exposure. All tables and figures are determined before any statistical procedure is undertaken, all tables will be filled in with results before the code for actual drug exposure is broken, and no information will be deleted when results are known.

### Ethical approval

The study protocol has been approved by the Regional Committee for Medical Research in Central Norway (2014/958).

### User involvement

No patients are involved in setting the research question or the outcome measures; nor are they involved in the design and implementation of the study. There are no plans to involve patients in dissemination.

### Access to data

There will be no additional data available.

### Dissemination policy

The study will give rise to a scholarly publication that will be published in an international, peer-reviewed journal.

## Discussion

In this article, we present a protocol for a nationwide study designed to investigate incidence rates of intracranial hemorrhage requiring hospitalization in users of different oral antithrombotic drugs. Treatment and outcome data are obtained from the Norwegian patient registry and the Norwegian prescription database. Secondary end-points are case-fatality rates at 3 months and 1 year after ICH, overall survival after ICH, and proportion of patients undergoing neurosurgical procedures in users and non-users of various antithrombotic drugs. In addition we will provide incidence rates for the different antithrombotic drugs in subgroups of ICH as they are defined in this study. ****

### Study strengths and limitations

The major strength of this study is the large nationwide sample size collecting real world data for antithrombotic medication and its relation to ICH. Further, the study is performed in a well-defined region, Norway, where patients have equal access to a public health care system and medications. This will assure results with high external validity. Another strength is the inclusion of subdural hematomas, which are often omitted from studies evaluating the incidence of ICH in patients on antithrombotic treatment (except when associated with high energy or penetrating trauma mechanism). We will also like to stress that all tables will be set in advance before any statistical procedure is undertaken, the statistical procedures will be performed by a statistician blinded to the actual antithrombotic treatment, all results will be filled in the tables before the codes for actual treatment are broken, and no information will be deleted when the results are known. By performing the study by these strict methodological measures we will as far as possible avoid presenting biased results.

The main limitation of our study is its observational design and lack of randomization. There is a lack of information about important clinical parameters including body mass index, blood pressure, tobacco use, lipid levels, and coagulation profile; hence the effect of unmeasured confounders cannot be excluded. Since data are partly based on diagnoses codes set in clinical practice, the results may be affected by the quality of coding practice.

There are tools available to estimate the risk of major bleeding for patients on anticoagulation treatment to help determine risk-benefit ^16^. Unfortunately, we have no data on the necessary variables in our patient population. Antithrombotic drugs are prescribed to patients with perceived higher risks of thrombosis. Some risk factors associated with thrombosis are also risk factors for ICH. Making causal inference from observed incidence rates of ICH in various categories of users and non-users of antithrombotic medications is difficult.

In large registry- and population-based pharmacoepidemiological studies some assumptions concerning drug exposure must be made. The ATC/DDD system has been in use for more than four decades in drug utilization studies. It has been found suitable for comparisons of drug utilization between different population groups and to monitor trends in drug use. Further, the ATC/DDD system is useful for providing denominator data for drug safety assessments. In everyday clinical practice individual patient characteristics and pharmacokinetic considerations must be accounted for. Consequently, the prescribed daily dose may differ from the DDD. Other researchers have estimated a daily drug exposure for the individual patient after comparing the accumulated drug dose and the elapsed time from consecutive prescriptions ^6^. Information about the prescribed daily dose is unfortunately not available in NorPD. However, the prescribed daily dose does not necessarily reflect actual drug doses consumed. All time measurements are from a reference date known only to NPR and NorPD. Prescriptions dispensed in the last few months of 2014 might extend beyond our study period. Some patients might therefore have had an undetected ICH in early 2015 with ongoing exposure. Moreover, only patients with ICH admitted to hospital are included in the present study. Together, these factors contribute to a more conservative risk estimate of ICH in users of antithrombotic medications. Further, there is likely to be some missing drug exposure in the first few months of the study period in 2008 as prescriptions were dispensed in 2007. This might increase the incidence rate of ICH in the control group without antithrombotic drug exposure.

Due to data privacy regulations all patient data are de-identified and we are not allowed to access patients electronic hospital records or diagnostic imaging to validate diagnoses and drug exposure. Validation of diagnoses and drug exposure, at least in a representative subgroup of patients, would probably strengthen our results.

## Conclusion

In this article, we present a protocol for a nationwide study designed to investigate incidence rates of ICH requiring hospitalization in users of different oral antithrombotic drugs. Treatment and outcome data are obtained from the Norwegian patient registry and the Norwegian prescription database. We want to perform this study by strict methodological measures and have discussed some of the methodological issues pertinent to the successful execution of this large-scale pharmacoepidemiological study.

## List of abbreviations

ATC = Anatomical Therapeutic Chemical

DDD = Defined Daily Dose

ICD-10 = 10 ^th^ revision of the International Statistical Classification of Diseases and Related Health Problems

ICH = Intracranial hemorrhage

ICPC-2 = version 2 of the International Classification of Primary Care

NorPD = Norwegian prescription database

NPR = Norwegian patient registry

RCT = Randomized controlled trials

STROBE = strengthening the reporting of observational studies in epidemiology
