# Supplementary material for: Risk of intracranial hemorrhage in users of oral antithrombotic drugs: Study protocol for a nationwide study
Source: F1000Res. 2015 Dec 30;4:1519. [Version 1] doi: 10.12688/f1000research.7633.1 (PMC4755390; doi:10.12688/f1000research.7633.1)
Supplement: Peer review history

# Peer review: Referee response for version 1

**DOI:** 10.5256/f1000research.8220.r12194

**Published:** 2016-02-15

**Authors:** Peter Siesjö

**Affiliations:** 1 Division of Neurosurgery, Department of Clinical Sciences Lund, Skåne University Hospital, Lund, Sweden

The present study outline by Gulati *et al. *aims to evaluate the incidence and relative risk of ICH after parenteral anti-thrombotic drug use. The aim is meritorious since very little data of these are known ^1^. Mostly the question of prolongation of anti-thrombotic therapy after ICH have been discussed ^2^. The presentation and discussion of the strengths and limitations of the proposed study is adequate and well organised. The only alternative to the present design would be a prospective trial but, given the limitations, the proposed retrospective study will be able to make a reasonable estimate of incidence and relative risks.

I have read this submission. I believe that I have an appropriate level of expertise to confirm that it is of an acceptable scientific standard.

## References

[1] FlynnRWMacDonaldTMMurrayGDDoneyAS: Systematic review of observational research studying the long-term use of antithrombotic medicines following intracerebral hemorrhage. *Cardiovasc Ther*.2010;28(3) : 10.1111/j.1755-5922.2009.00118.x 177-84 10.1111/j.1755-5922.2009.00118.x 20337638

[2] HofmeijerJKappelleLJKlijnCJ: Antithrombotic treatment and intracerebral haemorrhage: between Scylla and Charybdis. *Pract Neurol*.2015;15(4) : 10.1136/practneurol-2015-001104 250-6 10.1136/practneurol-2015-001104 25922539

# Peer review: Referee response for version 1

**DOI:** 10.5256/f1000research.8220.r11731

**Published:** 2016-01-18

**Authors:** Terje Sundstrøm, Christian A. Helland

**Affiliations:** 1 Department of Neurosurgery, Haukeland University Hospital, Bergen, Norway

This is a well-written and ambitious study proposal. Many patients are prescribed antiplatelet and anticoagulant therapies, and intracranial hemorrhage (ICH) is a particularly feared complication as it is associated with severe morbidity and mortality. These therapies are most frequently used in elderly people, and the extent of their use and adverse effects will be increasingly relevant, as the population is progressively getting older. This study aims to assess the risk of ICH in patients on different anti-thrombotic therapies in Norway. We agree with the authors that the reported incidence rates of ICH in such patients are probably lower than what is seen in regular clinical practice. Thus, it is of considerable interest to evaluate this in a real-world and heterogeneous population where these drugs are in use, and especially within a homogenous and transparent health system such as in Norway.

The study is generally well designed and methodologically sound. The described analyses will involve large groups of patients from the Norwegian Patient Registry and the Norwegian Prescription Database. However, we find the inclusion and exclusion criteria somewhat unclear and not in concordance with other sections of the manuscript. The first inclusion criterion is “hospitalization due to ICH”; this will enable the analysis of incidence rates of antithrombotic use in ICH patients. The most interesting incidence rates are those of ICH among all patients on these therapies, which will require inclusion of all Norwegian citizens on these drugs. These analyses are however described repeatedly throughout other sections of the protocol, and will give sound estimates of ICH incidence rates and a foundation for comparative analyses between different drugs and patient groups.

We also find the exclusion criteria slightly confusing. With the collected information, which specifically does not include detailed information from hospital journals, it will not be possible to exclude patients with “traumatic (high energy) intracranial injury “or “parenteral antithrombotic treatment”. On the other hand, it would be very interesting to evaluate the in-hospital incidence (and outcomes) of ICH in patients undergoing such treatments for stroke and myocardial infarction, but this is beyond the scope of this study.

The authors also want to assess the frequency of neurosurgical procedures in users and non-users of antithrombotic drugs. It is not clear which types of procedures this analysis will involve, but we suppose that craniotomies with hematoma evacuation, burr hole evacuation, ventricular drainage and intracranial pressure measurement are relevant procedures. The NOMESCO Classification of Surgical Procedures (NCSP) codes should be clearly described.

We believe that it will be difficult to differentiate between chronic and acute subdural hematomas (no specific ICD code for chronic or acute), as well as traumatic and non-traumatic hematomas (divergent practices using traumatic ICD code S06.5 and non-traumatic ICD code I62.0). One could deduce if the hematoma was chronic or acute through linkage analysis with the conducted neurosurgical procedures. However, it will be impossible to assess if it was chronic or acute in non-operated patients, or traumatic or non-traumatic, without detailed review of patient journals.

Taken together, this study protocol outlines a comprehensive and interesting study that can provide clinically relevant estimates of ICH risk in patients on antithrombotic drugs. In our opinion, the authors only need to provide minor clarifications to the study protocol.

We have read this submission. We believe that we have an appropriate level of expertise to confirm that it is of an acceptable scientific standard.
